# Mood and emotional reactivity of adolescents during the COVID-19 pandemic: short-term and long-term effects and the impact of social and socioeconomic stressors

**DOI:** 10.1038/s41598-021-90851-x

**Published:** 2021-06-02

**Authors:** Kayla H. Green, Suzanne van de Groep, Sophie W. Sweijen, Andrik I. Becht, Moniek Buijzen, Rebecca N. H. de Leeuw, Danielle Remmerswaal, Rianne van der Zanden, Rutger C. M. E. Engels, Eveline A. Crone

**Affiliations:** 1 Erasmus School of Social and Behavioural Sciences, Erasmus University Rotterdam, Burgemeester Oudlaan 50, 3000 DR Rotterdam, The Netherlands; 2 Research Center Adolescent Development, Utrecht University, Utrecht, The Netherlands; 3 Behavioural Science Institute, Radboud University Nijmegen, Nijmegen, The Netherlands

**Keywords:** Psychology, Psychology and behaviour

## Abstract

Adolescence is a formative period for socio-emotional development which is threatened by the COVID-19 pandemic. The current longitudinal study examined two aims: (1) the short- and long-term effects of the pandemic on young people’s mood (i.e. vigor, tension, and depression levels) and emotional reactivity (i.e. fluctuations in daily mood), and (2) the impact of stressors on mood, emotional reactivity, self-oriented (i.e. maladaptive behavior towards COVID-19 rules) and other-benefitting behaviors (i.e. behavior aimed at helping and comforting others). We conducted an online two-week daily diary study among 462 Dutch adolescents (*M*_age_ = 15.27 years, 64% females) and 371 young adults (*M*_age_ = 21.49 years, 81% females) in May 2020, with a follow-up in November 2020 (*N* = 238 and 231, respectively adolescents and young adults). In May 2020, young adults and older relative to younger adolescents showed higher levels and more fluctuations in tension and depression and lower levels of vigor. Vigor levels decreased and tension and depression levels increased between May 2020 and November 2020, especially for younger adolescents. There were positive associations between instability of negative emotions (i.e. tension and depression fluctuations) and the exposure to stressors (i.e. family stress and inequality of online homeschooling) in the adolescent sample. Together, this study demonstrates vulnerability regarding young people’s mood and emotional reactivity during the COVID-19 pandemic, especially for adolescents who experience more stressors.

## Introduction

Adolescence and young adulthood are formative periods for emotional and social development^1,2^. The COVID-19 pandemic threatens this development, as it required adolescents and young adults to deal with marked life changes like fully transitioning to online education, having limited to no physical contact with peers or friends, and being constrained to stay at home^3^. Both adolescents and young adults are likely to have experienced remarkable alterations in most of their social relationships, due to social distancing restrictions^4^.

The aim of this study is to test the emotional and social consequences of the COVID-19 pandemic and how these influence the self-oriented and other-benefitting behavior of individuals in these two formative phases for social development. While for some the COVID-19 pandemic might be a window of opportunity for positive other-benefitting behavior, such as helping and comforting, others may be prone to more self-oriented behaviors with possible negative consequences, such as not following societal rules^2^. The main aims of the current study are to: (1) test for age-related changes in daily levels and fluctuations of vigor, tension and depression early and later in the pandemic, and (2) test the pre-registered hypothesis that the interaction between emotional reactivity and COVID-19 related stressors shapes adolescents’ outcomes for better or worse, specifically concerning self-oriented and other-benefitting behaviors. In the present study, self-oriented behavior was operationalized as disobedient behavior with potentially harmful consequences and other-benefitting behavior was defined as behaviors related to helping and comforting others.

### Enhanced emotional reactivity during adolescence

An important determinant for individual differences in developmental outcomes is emotional reactivity^5^. Emotional reactivity can be seen as one’s emotional response to an event or changes in the environment^6^. An individual with higher levels of emotional reactivity typically shows stronger responsiveness to environmental influences. It has been well documented that adolescence is a period characterized by liable emotional states and increases in frequency and intensity of emotional reactivity^7,8^, with a peak during mid-adolescence causing heightened sensitivity to social and emotional changes^9–11^. This heightened adolescent emotional reactivity includes increased fluctuations, compared to childhood and adulthood, in both positive and negative emotions. In addition, across adolescence there is an increase in the experience of negative emotions and a decrease in the experience of positive emotions (i.e. the mean levels of emotions)^12^, but how these developmental patterns are affected by the COVID-19 pandemic is currently unknown. Although during young adulthood there is no longer a peak in enhanced emotional reactivity, there are still experiences of more frequent daily stressors, compared to older adults, which can have a negative influence on stress-related affect^13^.

Numerous studies have linked heightened emotional reactivity in adolescents to negative developmental outcomes such as risk-taking behavior, alcohol and substance abuse, and affective disorders^14–16^. However, this rise in emotional reactivity, along with an increased salience of peer relationships, also may serve an adaptive function for adolescents^8^. For example, recent neuroimaging studies have shown that ventral striatum reactivity, a neural indicator of reward, plays a role in the sensitivity to social connections, which in turn may stimulate other-benefiting behavior and healthy relationships^17^. In other words, this framework poses that increased emotional reactivity during adolescence can possibly also promote greater prosociality and help to successfully navigate challenging times, depending on whether adolescents grow up in a supportive or unsupportive environment. Therefore, an additional goal of this study is to shed light on the impact of the COVID-19 pandemic on young people’s mood and to use the abovementioned theoretical framework to predict under which circumstances heightened emotional reactivity aids or harms behavior during the pandemic.

### Social and socioeconomic stressors during the COVID-19 pandemic

Here we test emotional reactivity as a differential susceptibility marker for responding to challenges in the COVID-19 pandemic^18^. Depending on the specific stressors within the environment, higher emotional reactivity could lead to maladaptive behaviors in some individuals but in positive outcomes for others^19,20^. Adolescents may experience environmental stressors during the COVID-19 pandemic that interact with their emotional reactivity to impact their mental health. Environmental factors that are likely to impact adolescents’ mental health can be categorized in two types of stressors: social stressors and socioeconomic stressors.

Two important social stressors that are likely to impact adolescents’ mental health are family stress and emotional maltreatment. The COVID-19 outbreak has impacted some families more than others, which, in combination with adolescents being forced to spend more time at home with their parents and siblings, may threaten family wellbeing^21^. As a consequence, some adolescents have experienced more negative changes in their family- and social environment than others. It is important to acknowledge that there is heterogeneity in whether the increased time spent with family members during the first wave of the COVID-19 pandemic has positive or negative outcomes on both parents and adolescents. For example, some adolescents have experienced stress and tension within the family during the COVID-19 pandemic^22^, and heightened levels of stress within a family place some adolescents at risk for maltreatment^23^. A recent study on potential child abuse during the COVID-19 pandemic, indicated that parents with higher symptoms of anxiety and/or depression were at increased risk for maltreating their children^24^. Prior research has shown that the impact of chaotic circumstances in a household on a child’s wellbeing is influenced by the emotional reactivity of the child^25^. Children who grew up in a chaotic household and had a lower emotional reactivity showed fewer behavioral problems during adolescence, while higher emotional reactivity was related to more behavioral problems during adolescence^25^.

Besides social stressors, there are also socioeconomic stressors that may threaten adolescents’ mental health, such as limited educational opportunities and economic instability within households. A recent COVID-19 related study among Canadian adolescents revealed that 72% of the adolescents were very worried that the pandemic would impact their school year^26^. Issues related to proper internet connection or the lack of physical space to concentrate on schoolwork compose inequal opportunities for online home schooling and may contribute to experiences of stress. Moreover, some adolescents are confronted with sudden financial concerns within the family as the COVID-19 pandemic also has enormous economic consequences^4^. The same Canadian COVID-19 study also reported that 58% of the adolescents were a little or somewhat worried about their family’s finances, and 36% was very worried^26^. Although financial concerns could indeed negatively affect adolescent’s mood, it might also lead to other-benefiting behavior, especially to family members with whom they share these concerns^27^. Together, home schooling inequality and financial concerns in the family are socioeconomic stressors that may threaten adolescents’ mood, specifically during the COVID-19 pandemic.

### The present study

In the present study we tested the impact of the COVID-19 pandemic on young people’s mood, emotional reactivity, self-oriented and other-benefitting behavior. We conducted a two-week daily diary study during COVID-19 lockdown (two waves; May 2020 and November 2020) in which participants aged 10–25-years (i.e. adolescents and young adults) received online questionnaires every weekday, resulting in ten assessments per wave. We operationalized mood as mean levels of three emotions: vigor, depression, and tension, and operationalized emotional reactivity in terms of fluctuations in daily vigor, depression and tension. Self-oriented behavior was operationalized as disobedient behavior with potentially harmful consequences and other-benefitting behavior was defined as behaviors related to helping and comforting others. For this purpose, participants filled in questionnaires regarding their mood, stressors within the family context (i.e. family stress, emotional maltreatment, inequality of opportunity in online home schooling and financial concerns), the COVID-19 governmental rules (i.e. perceived unimportance of the rules, disobeying the rules) and societal commitment (i.e. emotional support to family, friends, and the willingness to help others during the pandemic), respectively. The aims of the present study were two-fold:

Aim 1 was to explore the mean levels and fluctuations of *vigor, tension, and depression*, to unravel state specific effects for mood and emotional reactivity among two age samples: adolescents and young adults, at two time points: May 2020 (T1) and November 2020 (T2). We also explored whether mood and emotional reactivity showed age-related differences across adolescence and young adulthood. We conducted a follow-up two-week daily diary study in November 2020, to assess the long-term effects of the COVID-19 pandemic on the mean levels and fluctuations of the three mood states (preregistration: https://osf.io/d62y7/) and to explore whether changes in mood and emotional reactivity were age-dependent. We expected a decrease in vigor mean levels and an increase in mean levels of tension and depression^28,29^. It was hypothesized that fluctuations in all three mood states would increase between May 2020 (T1) and November 2020 (T2)^30^.

Aim 2 was to test the hypotheses that the associations between heightened emotional reactivity and adolescents’ self-oriented and other-benefitting behaviors are moderated by social and socioeconomic stressors within the family context (preregistration: https://osf.io/uf9dn/). Consequently, aim 2 is restricted to the adolescent sample, as the young adult sample consisted of college students who were less likely to live with their family. Aim 2 was solely tested using data from May 2020 (and not November 2020) for several reasons. First, we aimed to specifically test the effect of social and socioeconomic stressors during the period in which young people could not attend schools and were forced to spend more time within the family context, which was the case in May 2020 but not November 2020. Second, moderation analyses to examine the effects of individual differences require relatively large sample sizes for adequate statistical power, which is another reason why the data acquired in May were more suitable to answer the second research aim (*N*_*May*_ = 462, *N*_*No*v_ = 238).

We examined pre-registered associations between emotional reactivity and self-oriented and other-benefitting behavior. We expected no main effects of mood fluctuations (i.e. vigor, tensions, and depression) on self- (i.e., perceived unimportance of the rules, disobeying the rules) and other-benefitting behavior (i.e., emotional support to family and friends, and the willingness to help others during COVID-19). Instead, we expected interaction effects with social and economic stressors. We expected that emotional reactivity would serve as a differential susceptibility factor. That is, we tested whether individuals with higher levels of emotional reactivity were more likely to show negative outcomes, such as harmful self-oriented behavior, compared to individuals with lower levels of emotional reactivity when in a family environment in which they are dealing with social and socioeconomic stressors. At the same time, we expected that when in an environment with less exposure to social and socioeconomic stressors, individuals with higher levels of emotional reactivity will show positive outcomes, such as prosocial other-oriented behavior, compared to individuals who are less emotionally reactive.

## Methods

### Participants

Two samples participated: an adolescent and a young adult sample. With regard to the adolescent sample, 511 adolescents initially applied for the present study, of whom 485 actually participated. Of these 485 adolescents, 23 adolescents were excluded because they reported mood states on less than three days. Hence, the final sample included 462 adolescents (*M*_age_ = 15.27 years, *SD* = 1.79, age range 10–20 years, 64% females) at the first timepoint. 431 of the 485 adolescents gave permission to be invited for the second timepoint of the study in November 2020. In total 258 adolescents reapplied for the second wave of the present study, of whom 255 also participated. 17 adolescents were excluded as they reported mood states on less than three days. The final adolescent sample consisted of 238 participants (*M*_ageT1_ = 15.54 years, *SD*_T1_ = 1.77, age range_T1_ 10–19 years, 75% females).

In the young adult sample 473 college students applied, 463 of whom also participated. We recruited college students specifically as they were in a comparable situation as the adolescents being restricted to attend education. We excluded 22 participants as they were not college students. Additionally, 37 participants were excluded for being older than 25 years (i.e., they did not fit our pre-defined young adult age range) and 33 participants were excluded for not having three or more days of mood state measurements. This resulted in a final sample of 371 young adults (*M*_age_ = 21.49 years, *SD* = 1.91, age range 17–25, 81% females). Those young adults who attended a university programme and who gave permission for follow-up requests were invited for the second wave (N = 404). Initially, 266 young adults participated in the second wave of the present study. We excluded 10 participants as they reported mood states on less than three days, and 25 for not fitting our predefined age range at T1 (i.e. 17–25 years). In the final sample 231 young adults were included (*M*_ageT1_ = 21.26 years, *SD*_T1_ = 1.89, age range_T1_ 17–25 years, 84% females). Table 1 shows detailed information on the sample compositions. The adolescent sample was representative of the Dutch population in terms of ethnicity (i.e. 25% of the current sample did not exclusively identify as Dutch, nationwide this is 28%)^31^. However, in terms of educational level, both the adolescent and young adults sample were attending above average education levels^31^.

**Table 1:** Overview on the sample characteristics for the adolescents (N = 462) and the students (N = 371) in May 2020 (T1).

	Adolescents	Students
Variable	N (percentage of sample)	N (percentage of sample)
**Age (years)**		
10–12	46 (10.0)	0
13–15	250 (54.1)	0
16–18	163 (35.3)	31 (8.4)
19–21	3 (0.6)	189 (50.9)
22–24	0	139 (37.5)
25	0	12 (3.2)
**Educational level**		
Elementary school	8 (1.7)	0
Pre-vocational education	11 (2.4)	0
Higher general continued education	152 (32.9)	0
Preparatory scientific education	274 (59.3)	0
Vocational education	1 (0.2)	0
College education	1 (0.2)	0
Academic education	1 (0.2)	371 (100)
**Parental educational level**		
Low	42 (9.1)	71 (19.1)
Medium	141 (30.5)	147 (39.6)
High	196 (42.4)	136 (36.7)
**Birth country**		
Netherlands	440 (95.2)	246 (66.3)
Other European country	5 (1.1)	70 (18.9)
Other country outside Europe	6 (1.3)	53 (14.3)
**Ethnicity**		
Dutch	335 (72.5)	200 (53.9)
Non-Dutch	15 (3.2)	113 (30.5)
Multiple ethnicities, including Dutch	97 (21.0)	49 (13.2)
Multiple ethnicities, all non-Dutch	3 (0.6)	6 (1.6)
**Pre-COVID living situation**		
With (foster) parent(s)	447 (96.8)	106 (28.6)
With sibling	1 (0.2)	2 (0.5)
With partner	0	25 (6.7)
Alone	2 (0.4)	43 (11.6)
With roommates	0	193 (52.0)
Changed living situation during COVID-19 pandemic (yes)	47 (10.2)	137 (36.9)
**Mental/neurological disorder**		
Current	36 (7.8)	48 (12.9)
Past	43 (9.3)	74 (19.9)

Percentages do not necessarily add up to 100, due to missing data. Responses with “I do not know” were reported as missing data.

We explored potential differences between the attrition and non-attrition group. We performed two separate one-way ANOVAs, per age sample, to assess whether the attrition group differed from the non-attrition group on vigor, tension, and depression mean levels and fluctuations during T1. The results revealed no significant differences, indicating that the mood levels and mood fluctuations at T1 where similar between the attrition and non-attrition group (ps > 0.10).

### Procedure

For the recruitment of adolescents, we reached out to various secondary and high schools in the Rotterdam area in the Netherlands through existing contacts with the university. All adolescents from participating schools received a notification about our study through their schools communication platform. Those who were interested in participation could subscribe via an online form. Inclusion criteria were: living in the Rotterdam area and being aged 10 – 22 years. The young adults were university students who attended a college program at Erasmus University Rotterdam (EUR). They were approached through the EUR website, email and social media platforms. Inclusion criteria were: attending EUR (whether as a national or international student) and being aged 17–25 years. Participants received a monetary reward of €15 for their participation, regardless of the amount of completed questionnaires. Participants gave permission by means of informed consent. In line with the guidelines from the ethics committee, parental informed consent was ensured for participants aged 15 years or younger. The present study was approved by the ethics committee of the Erasmus School of Social and Behavioural Sciences at the EUR (application 20-036) and conducted in accordance with the guidelines and regulations from the ethics committee.

Participants received daily online questionnaires for two consecutive weeks from Mondays to Fridays, resulting in ten questionnaire days in total, in both May 2020 and November 2020. Data were collected using survey software platform Qualtrics. Based on their application date participants were assigned to two different batches in May: batch one starting on May 4, 2020 and batch two on May 11, 2020. Participants who applied on May 4th or later were assigned to batch two. In November 2020 participants were invited in one single batch. The COVID-19 related restrictions during May 2020 and November 2020 in the Netherlands were: social distancing (1.5 m physical distance), no large gatherings in groups, limited visitors allowed at home, staying home in case of symptoms, and closing of high schools and universities (latter applied to May 2020 only, however in November 2020 there were signals of re-closing the schools).

Daily invitations to fill out the questionnaire were sent by email at 12:00 AM. In addition, a text message was sent at 19:00 PM as a reminder to participants who had not yet filled in the questionnaire of that particular day. On day 1, 5 and 10 the duration of the questionnaires was 15–20 min due to extra measures. All other days the duration was 5 to 10 min. Participants were instructed to fill out the questionnaire on the day of the invitation. They were also encouraged to respond to the new invitations even after missing one or more days. See S1 for an overview of the data preparation steps.

### Measures

#### Mood and emotional reactivity

The shortened Dutch translation of the Profile of Mood States Scale (POMS) was used to assess *mood levels* and *mood fluctuations*^32^. For the present study, we focused on three subscales: vigor (five items, Cronbach’s α_Day1_ = 0.77 and 0.80 for adolescents on respectively T1 and T2; Cronbach’s α_Day1_ = 0.84 and 0.84 for young adults), tension (six items, Cronbach’s α_Day1_ = 0.84 and 0.86 for adolescents on respectively T1 and T2; Cronbach’s α_Day1_ = 0.86 and 0.87 for young adults), and depression (eight items, Cronbach’s α_Day1_ = 0.91 and 0.91 for adolescents on respectively T1 and T2; Cronbach’s α_Day1_ = 0.90 and = 0.92 for young adults). Participants were instructed to indicate to what extent they felt that the descriptions represented their current mood state. The questionnaire used a five-point Likert scale ranging from 1 (“not at all”) to 5 (“extremely”). Mean scores were computed for each subscale as a measure of mood level. Mood fluctuations for each subscale were measured using the within person standard deviation approach^33^.

#### Social stressors

We examined two types of social stressors: family stress and emotional maltreatment. *Family stress* was assessed with two items of the recently developed Pandemic Questionnaire (van de Groep et al., https://osf.io/kgcdm/) (Cronbach’s α_Week1_ = 0.70): “I think my family and I live too much in each other’s pockets since the schools are closed and most people work from home” (1) and “I would rather be as little as possible at home, since there is too much tension going on” (2). This selection of the Pandemic Questionnaire used a seven-point Likert scale, ranging from 1 (“not at all”) to 7 (“totally true”). The items were administered weekly. A mean score was computed of the two items of both weeks.

*Emotional maltreatment* was assessed as a lifetime experience using the Childhood Trauma Questionnaire-Short Form, a questionnaire proven to be reliable and valid among adolescents and adults^34^. In the present study, we used the emotional neglect (5 items, Cronbach’s α_Week1_ = 0.84) subscale and a selection of the emotional abuse items (3 items, Cronbach’s α_Week1_ = 0.61), including, “I feel loved”, “People in my family look out for each other” (both reversed scored), “I feel that someone in my family hates me”, and “People in my family say hurtful and insulting things to me”. The questionnaire used a five-point Likert Scale, ranging from 1 (“(almost) never”) to 5 (“(almost) always”). We combined the items of the two subscales and computed standardized mean scores. The questionnaire was administered weekly.

#### Socioeconomic stressors

The Inequality of Opportunity in Home schooling Questionnaire developed for the Covid-19 pandemic (Green et al., https://osf.io/9g8cq/) was used to assess *inequality of opportunity with regard to home schooling during the COVID-19 pandemic crisis*. The questionnaire consisted of five items (Cronbach’s α_Week1_ = 0.68) and used a five-point Likert scale ranging from 1 (“not at all”) to 5 (“totally true”). Participants were asked if they were dealing with difficulties in online home schooling. They were requested to indicate to what extent situations such as: “stable internet connection in order to take online classes”, “a room in the house where you can do your schoolwork”, and “a proper functioning laptop/computer/tablet in order to take online classes” applied to them. The questionnaire was administered weekly. Mean scores were computed.

A single-item from the Pandemic Questionnaire (van de Groep et al., https://osf.io/kgcdm/) was used to assess *financial concerns within the family due to the COVID-19 pandemic crisis*, namely: “Are you worried that there will be financial problems in your family due to the COVID-19 pandemic?”. The Pandemic Questionnaire consisted of open and multiple-choice questions that were specifically targeted to measure contributions to selfish behavior and risk behavior during COVID-19 pandemic. The selected item had a response scale from 1 (“never”) to 5 (“almost always”) and was administered weekly. We computed the mean score of the week 1 and 2.

#### Self-oriented behavior

Self-oriented behavior in adolescents was operationalized as the unwillingness to follow the advised protective rules. We used a selection from the Pandemic Questionnaire (van de Groep et al., https://osf.io/kgcdm/). The items were grouped into two subscales: *perceived unimportance of the rule*s (3 items, Cronbach’s α_Week1_ = 0.63), including items like “I would at this moment go to a party if my friends were organizing one at home”. This subscale had a seven-point Likert scale, ranging from 1 (“entirely disagree”) to 7 (“entirely agree”). Responses were reversed, hence a higher score represents more perceived unimportance of the rules. The *disobeying the rules* (2 dichotomous items) subscale contained items like “Did you meet with a friend last week while you were coughing, experiencing a sore throat, a runny nose, or some other health complaint?”. The items were administered weekly. For both subscales mean scores were computed.

#### Other-benefitting behavior

Other-benefitting prosocial behavior was conceptualized as: emotional support to family, to friends, and the willingness to help others during COVID-19. We used two measures from a prior study to assess these three forms of prosocial behavior^35^. An adapted subscale from the Opportunities for Prosocial Actions questionnaire was used to assess *emotional support to family and friends* during the COVID-19 pandemic. Per target (i.e. friend and family member, respectively Cronbach’s α_Day1_ = 0.74 and Cronbach’s α_Day1_ = 0.74) the following three items were administered: “I comforted a family member today”, “Today I did my best to spend my time with family”, and “Today, I called and/or send a message to a family member”. Responses were given on a scale from 0 (“not at all”) to 5 (“a lot”). Mean scores were computed per target. The items were administered daily.

The Contributions to Society questionnaire was used to *assess the willingness to help others during the COVID-19 pandemic*. Three items from this questionnaire were adapted to be suitable to the current COVID-19 pandemic crisis (Cronbach’s α_Day1_ = 0.74): “I have been committed to society for the past 24 h”, “I have helped others in the past 24 h”, and “I've been working for the people around me for the past 24 h”. Responses on the statements were given on a seven-point Likert scale ranging from 1 (“not at all”) to 7 (“totally true”). The items were administered daily. Mean scores were computed.

## Results

### Aim 1: unravel the short term and long-term effects of COVID-19 on mood and emotional reactivity

Both the adolescent and student sample from May 2020 (N_T1_ = 833) and November 2020 (N_T2_ = 469) were included in Aim 1, in which we explored the initial and long-term impact of the COVID-19 pandemic on young people’s mood and emotional reactivity. In addition, we explored whether the exposure to social and socioeconomic stressors changed over time between T1 and T2.

#### Differences in mood states and age-related effects at T1

**Figure 1:**
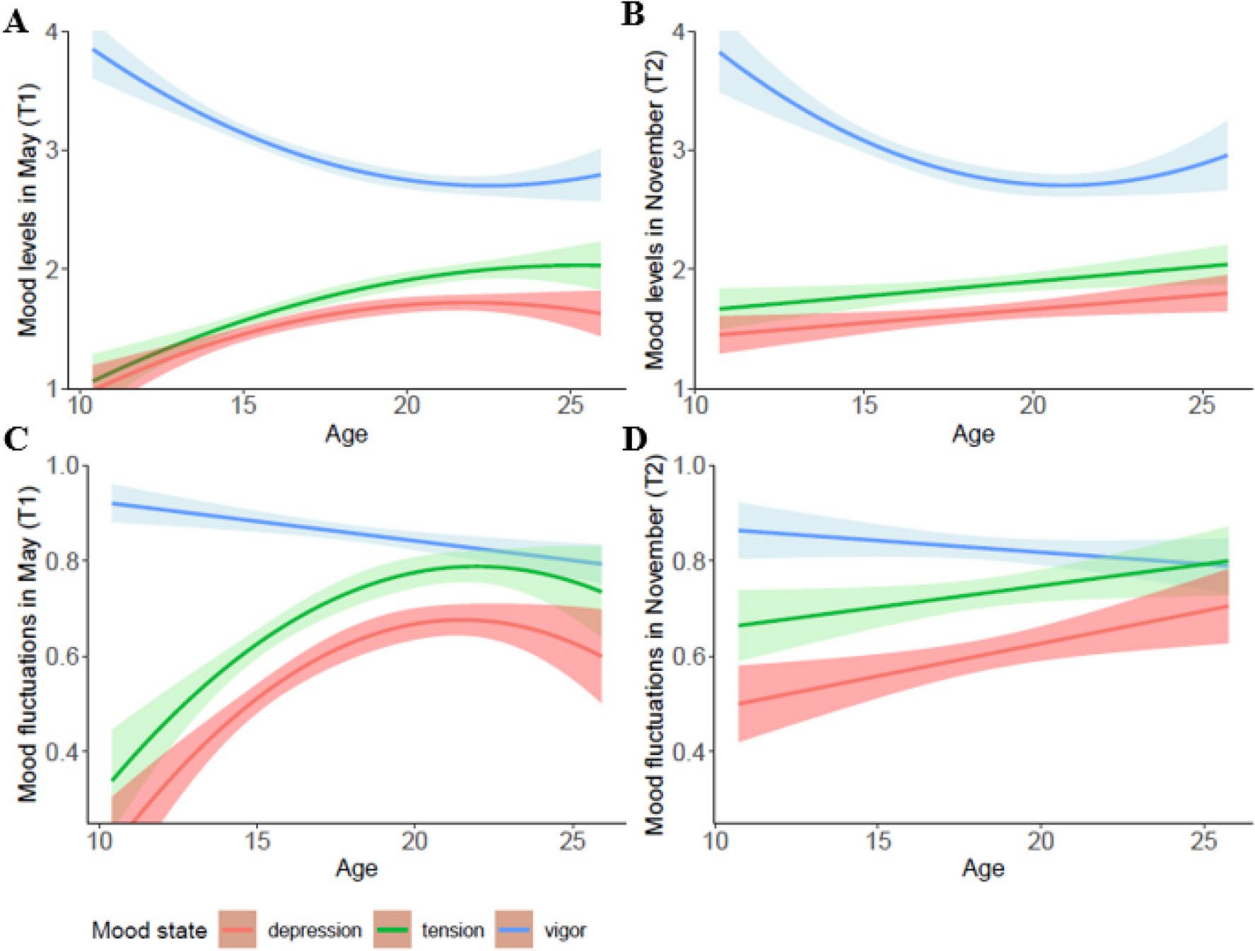
Graph A: adolescents and young adults reported higher levels of vigor compared to tension and depression in May 2020 (T1). All three mood levels showed quadratic age effects, with a drop in vigor and a peak in tension and depression in young adulthood. Graph B: adolescents and young adults reported higher levels of vigor compared to tension and depression in November 2020 (T2). Vigor showed a quadratic age effect with a drop in young adulthood, whereas tension and depression mean levels showed positive linear effects. Graph C: Adolescents and young adults reported more fluctuations in vigor, compared to tension and depression, which was specifically pronounced among younger adolescents at T1. Fluctuations in vigor showed a negative linear effect as a function of age, while tension and depression fluctuations peaked during late adolescence/young adulthood. Graph D: Adolescents show more vigour fluctuations compared to tension and depression fluctuations at T2. This disparity is less profound among young adults. There was no age effect of vigour fluctuations. However, tension and depression fluctuations showed a positive linear age effect across adolescence.

Assumption checks and correlations among variables can be found in S2. Separate repeated measures ANOVAs were performed to explore (1) differences in mean levels of the three mood states and (2) differences in mood fluctuations. The first results showed a main effect of mood mean level, *F* (1.16, 966.30) = 236.99, *p* < 0.001, *η*^*2*^ = 0.22. To test how these mood states differed across age, linear and quadratic age were entered to the model in a stepwise manner as continuous predictor. The results showed a significant linear age × mood state interaction effect, *F* (1.16, 966.30) = 94.52, *p* < 0.001, *η*^*2*^ = 0.10, as well as a significant quadratic age × mood state interaction effect, *F* (1.17, 967.34) = 14.09, *p* < 0.001, *η*^*2*^ = 0.02. Follow up analyses revealed that the quadratic models explained significantly more variance in the levels of the three mood states than the linear models (vigor *ΔR*^*2*^ = 0.02, *p* < 0.001; tension *ΔR*^*2*^ = 0.01, *p* = 0.026; and depression *ΔR*^*2*^ = 0.01, *p* = 0.003). As can be seen in Fig. 1a, there was a quadratic age effect for vigor, *F* (2, 830) = 45.55, *p* < 0.001, showing that vigor decreased with increasing age after which it reached a plateau in early adulthood, and a quadratic age effects for tension (*F* (2, 830) = 42.67, *p* < 0.001) and depression, *F* (2, 830) = 23.44, *p* < 0.001, showing that depression and tension increased with increasing age after which it reached a plateau in early adulthood.

Next, we tested differences in mood fluctuations. There was a main effect of mood fluctuation, *F* (1.71, 1418.89) = 123.09, *p* < 0.001, *η*^*2*^ = 0.13. Pairwise comparisons with Bonferroni correction revealed more fluctuations in vigor (*M* = 0.86, *SD* = 0.25), compared to tension (*M* = 0.69, *SD* = 0.35) and depression (*M* = 0.58, *SD* = 0.35), and more fluctuations in tension than in depression (Fig. 1c).

Significant interaction effects were found first for linear age × mood fluctuation, *F* (1.71, 1418.89) = 66.01, *p* < 0.001, *η*^*2*^ = 0.07) and then for quadratic age × mood fluctuation, *F* (1.72, 1429.27) = 14.88, *p* < 0.001, *η*^*2*^ = 0.02). Post hoc analyses revealed a negative linear effect of age on vigor fluctuations, *F* (1, 831) = 11.91, *p* = 0.001, vigor fluctuations decreased with age. The quadratic model did not explain additional significant variance in vigor fluctuations. In contrast to vigor, adding quadratic age to the model did result in greater explanation of the variance in tension and depression fluctuations (tension *ΔR*^*2*^ = 0.02, *p* < 0.001; depression *ΔR*^*2*^ = 0.02, *p* < 0.001). We found quadratic effects of age on tension, *F* (2, 830) = 34.48, *p* < 0.001, and depression, *F* (2, 830) = 35.76, *p* < 0.001, fluctuations, with a peak in early adulthood around age 20 (Fig. 1c).

#### Differences in mood states and age-related changes at T2

To examine whether the T1 (i.e., May 2020) mood patterns would be replicated at T2 (i.e., November 2020), we performed repeated measures ANOVAs. For T2, like T1, we found a main effect of mood state, *F* (1.16, 539.24) = 60.36, *p* < 0.001, *η*^*2*^ = 0.11. Adding age, both as a linear and quadratic factor, to the model resulted in significant interaction effects, *F* (1.16, 539.24) = 18.5, *p* < 0.001, *η*^*2*^ = 0.04, and *F* (1.16, 539.06) = 6.38, *p* = 0.009, *η*^*2*^ = 0.01 (respectively linear and quadratic). Post-hoc analyses revealed that compared to the linear model, the quadratic model explained significantly more of the variance in vigor (*ΔR*^*2*^ = 0.03, *p* < 0.001). As can be seen in Fig. 1b, we found that vigor levels increased during adulthood after an initial dip during late adolescence/early adulthood, *F* (2, 466) = 18.93, *p* < 0.001. Furthermore, we found a positive linear age effect for mean levels of tension during the second wave, *F* (1, 467) = 5.70, *p* = 0.017. Adding the quadratic age factor did not lead to an increase in the explained variance in tension (*p* > 0.05). Finally, the results showed a positive linear age effect for depression, showing an increase in mean levels across adolescence, *F* (1, 467) = 5.84, *p* = 0.016 (see Fig. 1b). Again, adding the quadratic age factor did not lead to an increase in the explained variance in tension (*p* > 0.05). In sum, the mood mean level results at T2 overall replicate the findings at T1.

Next, we tested the difference in mood fluctuations at T2. We found a main effect of mood state, *F* (1.76, 821.85) = 22.50, *p* < 0.001, *η*^*2*^ = 0.05. We found an interaction effect with age as linear factor, *F* (1.76, 821.85) = 8.98, *p* < 0.001, *η*^*2*^ = 0.02. Adding age as quadratic factor to the model did not result in a significant interaction effect (*p* > 0.05). Post-hoc tests, revealed no linear age effect in vigor fluctuations (*p* > 0.05). We found positive linear effects of age on tension, *F* (1, 467) = 3.94, *p* = 0.048, and depression fluctuations, *F* (1, 467) = 7.82, *p* = 0.005 (see Fig. 1d). Overall, mood fluctuation levels in November 2020 partially overlap with the pattern found in May 2020. That is adolescents showed more fluctuations in vigor, at both time points, but in November 2020 the peak in negative emotions was no longer present.

#### Long-term effects of COVID-19 on mood and emotional reactivity

To test the hypothesis that the continuation of the COVID-19 pandemic has a negative impact on adolescents’ mood and emotional reactivity, we performed repeated measures ANOVAs to assess effects of time on mood mean levels and fluctuations. In both models, we added linear and quadratic age as continuous predictor in a stepwise manner to test for covariate effects. Post-hoc analyses included linear and curve estimation regression analyses per outcome (i.e. vigor, tension, and depression). Of the 494 adolescents and young adults at T2, 35 participated at T1 but were later excluded due to an insufficient number of mood state reports (< 3 days). Hence, we included measurements at both time points for 459 participants in total. The analyses described below were performed within this sample.

We found a main effect of time, *F* (1.00, 457.00) = 7.45, *p* = 0.007, *η*^*2*^ = 0.02, and an interaction effect between time (i.e. T1 vs. T2) and mood state, *F* (1.27, 578.59) = 25.82, *p* < 0.001, *η*^*2*^ = 0.05. Post-hoc tests revealed that there was a decrease in vigor levels from T1 (*M* = 2.96, *SD* = 0.78) to T2 (*M* = 2.90, *SD* = 0.79), *t* = 2.32, *p* = 0.021. In contrast, we found increases in tension (*M*_*T1*_ = 1.74, *SD*_*T1*_ = 0.72; *M*_*T2*_ = 1.85, *SD*_*T2*_ = 0.77) and depression levels (*M*_*T1*_ = 1.56, *SD*_*T1*_ = 0.65; *M*_*T2*_ = 1.63, *SD*_*T2*_ = 0.71) between T1 and T2, respectively *t* = − 4.37, *p* < 0.001 and *t* = − 2.75, *p* = 0.006.

We also found an interaction effect between time, mood state, and linear age, *F* (1.27, 578.59) = 20.05, *p* < 0.001, *η*^*2*^ = 0.04. The decrease in vigor levels and the increases in tension and depression levels between T1 an T2 were greater among the younger compared to older adolescents/young adults, see Fig. 1a,b. There was no interaction with age as quadratic factor (*p* > 0.05).

The same analysis was performed for changes in mood fluctuations over time. We found a main effect of time, *F* (1.00, 865.78) = 7.45, *p* = 0.002, *η*^*2*^ = 0.02, which was qualified by an interaction effect between time and mood state, *F* (1.89, 578.59) = 6.87, *p* = 0.001, *η*^*2*^ = 0.02. Post-hoc tests revealed that there was a trend towards a decrease in vigor fluctuations between T1 (*M* = 0.85, *SD* = 0.24) and T2 (*M* = 0.82, *SD* = 0.26), *t* = 1.94, *p* = 0.053. We found increases in tension fluctuations between T1 (*M* = 0.70, *SD* = 0.34) and T2 (*M* = 0.73, *SD* = 0.33), *t* = − 2.23, *p* = 0.027 There were no significant differences in depression fluctuations between T1 and T2 (*p* > 0.05).

Finally, we found an interaction effect between time, mood state, and linear age, *F* (1.89, 865.78) = 4.90, *p* = 0.009, *η*^*2*^ = 0.01, such that the increase in tension fluctuations between T1 an T2 was greater among the younger adolescents, see Fig. 1c,d. There was no significant interaction effect with age as quadratic factor (*p* > 0.05).

### Aim 2: examine emotional reactivity as susceptibility marker within the family context

For Aim 2 we used the May 2020 measurements of the adolescent sample (N = 462) to examine the effects of family household experiences and emotional reactivity on self- and other-benefitting behaviors.

#### Associations between emotional reactivity and stressors

Assumption checks on all measures as well as a complete overview on the correlations between all independent and dependent variables can be found in S3. To investigate the relations between emotional reactivity and the social and socioeconomic stressors, we performed 15 partial correlational analyses. Multiple tests may lead to an increase in Type I error, therefore we used Bonferroni correction for multiple comparisons, *p* = 0.003^36,37^. All analyses were controlled for mean mood levels.

**Figure 2:**
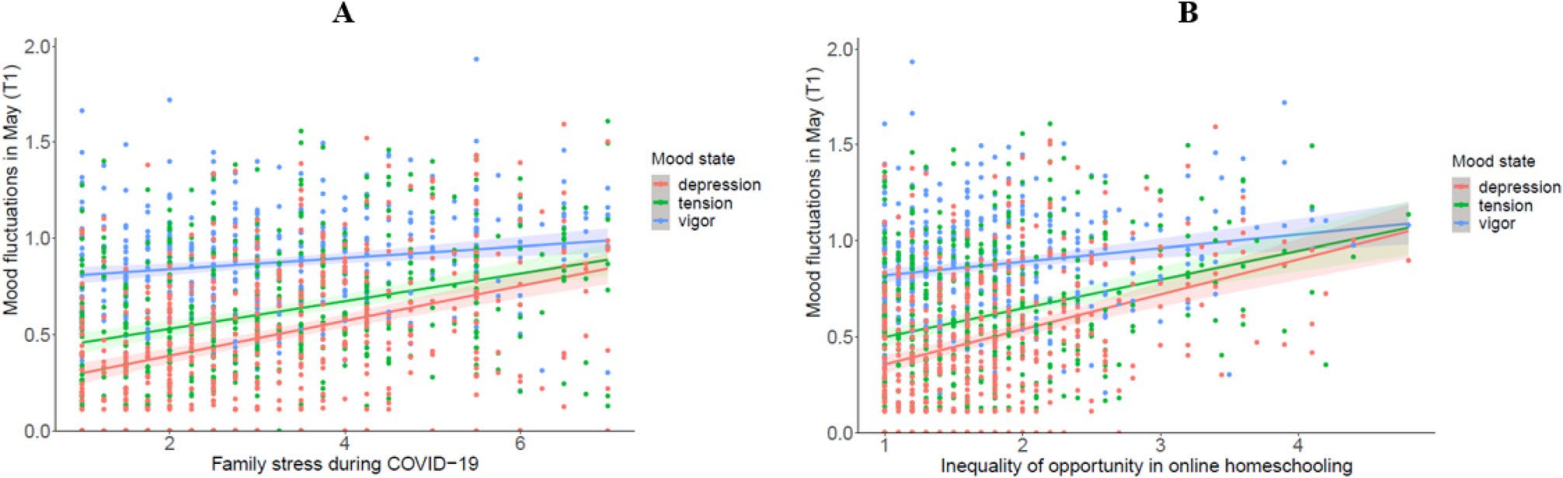
Graph A illustrates the positive associations between family stress and tension and depression fluctuations among adolescents in May 2020 (T1) (N = 462). Graph B illustrates the positive association between the inequality of opportunities in online home schooling and depression fluctuations among adolescents at T1.

We found no significant associations between vigour fluctuations and the social and socioeconomic stressors. For tension fluctuations, the findings showed a positively association with experiences of family stress (*r* = 0.16, *p* = 0.001), see Fig. 2a. With regard to depression fluctuations, we found significant positive association with family stress (*r* = 0.19, *p* < 0.001) and the inequality of opportunity in online home schooling (*r* = 0.19, *p* < 0.001). As can be seen in Fig. 2b, adolescents with more exposure to these stressors reported more fluctuations in their feelings of depression.

#### Associations between emotional reactivity and self-oriented and other-benefitting behaviors

To test the effect of emotional reactivity on self-oriented and other-benefitting behaviors, we performed hierarchical regression analyses in which fluctuation in vigor, tension, and depression were entered as independent variables, and the opportunity to provide emotional support to family and friends, the willingness to help others during the pandemic (i.e. benefitting-oriented behavior), the perceived unimportance of the rules, and disobeying the rules (i.e. self-oriented behavior) as dependent variables. To control for mood, mean levels were entered in a second step in each relevant model. We performed hierarchical regression analyses per mood fluctuation (vigor, tension, and depression) and dependent variable (five measures of self-oriented and other-benefitting behaviors), resulting in 15 regression analyses in total. We only report effects that survived Bonferroni corrections, resulting in p < 0.01, controlling for dependency between dependent measures^36,37^. The independent variables were mean centered prior the analyses in order to diminish multicollinearity when testing main effects^38^.

The results revealed no main effects of fluctuations in vigor, tension, and depression on the perceived unimportance of the rules, disobeying the rules, the opportunity to provide emotional support to family and friends, and the willingness to help others during the COVID-19 pandemic (*all ps* > 0.05). Detailed information on the associations between the social and socioeconomic stressors and self-oriented and other-benefitting behaviors can be found in S4.

#### Interaction effects between emotional reactivity and social and socioeconomic stressors on self-oriented and other-benefitting behaviors

The macro PROCESS by Hayes was used to perform moderation analyses in order to test for interaction effects between the social and socioeconomic stressors and emotional reactivity (i.e. mood fluctuations) on self-and other-oriented behaviors^39^. Correlation analyses revealed moderate to high correlations between the measures of each stressor type, respectively *r* = 0.51 and *r* = 0.40 for the social and socioeconomic stressors (see Table S3b). We, therefore, combined emotional maltreatment and family stress to create a social stressor composite score, and inequality of online home schooling and financial concerns to create a socioeconomic composite score. Composite scores were computed by calculating the mean of the standardized z-scores. This section includes multiple tests, we therefore only report effects that survived Bonferroni corrections, for multiple comparisons with an alpha of < 0.002^36,37^.

No effects were found with regard to self-oriented behaviors (i.e., perceived unimportance of the COVID-19 rules and disobeying the COVID-19 rules) and emotional support to family (see S5ab). Next, we assessed the hypothesis that social and socioeconomic stressors moderated the associations between mood fluctuations and the opportunity to provide emotional support to friends. Results did not survive the Bonferroni correction, and hence none of the effects were significant (see Table 2 for all p-values that did not survive the correction, see Table S5ab for all other non-significant findings). Lastly, we tested the hypothesis that social and socioeconomic stressors moderate the associations between mood fluctuations and the willingness to help others during COVID-19. As can be seen in Table 2, no significant effects were found as the findings did not survive the Bonferroni correction.

**Table 2:** Interaction effects between emotional reactivity and stressors on self-oriented and other-benefitting behaviors among adolescents in May 2020 (T1) that did not survive the Bonferroni correction.

	*t*	*b*	*SE*	*p*
**Self-oriented behaviour**				
Perceived unimportance COVID-19 rules	N.S	N.S	N.S	N.S
Disobeying the COVID-19 rules	N.S	N.S	N.S	N.S
**Other-benefitting behavior**				
Emotional support to family	N.S	N.S	N.S	N.S
Emotional support to friends				
Vigor fluctuations × social stressor	1.98	0.60	0.30	0.048
**Helping others during COVID-19**				
Vigor fluctuations × social stressor	2.37	0.79	0.33	0.018
Tension fluctuations × social stressor	− 2.36	− 0.54	0.23	0.019
Vigor fluctuations × socioeconomic stressor	2.64	0.82	0.31	0.009

The social stressor is a composite score of the measures emotional maltreatment and family stress, the socioeconomic stressor is a composite score of the measures inequality of opportunity in online home schooling and financial concerns.

#### Long-term effects of COVID-19 on social and socioeconomic stressors

For descriptive exploratory purposes we examined whether the social and socioeconomic stressors changed over time. We performed paired sample t-tests, per variable, to test for changes over time. We found an increase in exposure to emotional maltreatment by family members between T1 (*M* = 1.72, *SD* = 0.65) and T2 (*M* = 1.84, *SD* = 0.76), *t* = − 3.51, *p* = 0.001. At the same time, adolescents reported overall a decrease in family stress between T1(*M* = 3.32, *SD* = 1.64) and T2 (*M* = 3.06, *SD* = 1.65), *t* = 2.54, *p* = 0.012. No significant changes over time were found for inequality of opportunity online home schooling and financial concerns (*ps* > 0.05).

## Discussion

In the present study we investigated (1) the impact of the COVID-19 pandemic on adolescents’ and young adults’ daily mood during the first months of the crisis, and (2) whether mood states differed across adolescence and young adulthood (i.e., as a function of age), (3) long-term effects of the pandemic on daily mood and mood fluctuations, and (4) the moderating role of social and socioeconomic stressors on the associations between emotional reactivity and self-oriented and other-benefitting behaviors among adolescents. During both the first and second wave (May and November 2020) of the pandemic in the Netherlands, adolescents and young adults showed higher levels of vigor compared to tension and depression, along with more fluctuations in their daily vigor levels. In May 2020, these differences between mood states were smaller in older adolescents, but from May 2020 to November 2020 these differences particularly decreased for younger adolescents (i.e., they were most sensitive to mood changes over time). A closer examination of how heightened emotional reactivity during adolescence interacts with COVID-19-induced social and socioeconomic stressors, revealed no evidence that emotional reactivity acts as a differential susceptibility marker. We did find associations between tensions and depression fluctuations on the one hand, and the social and socioeconomic stressors on the other hand.

On average adolescents and young adults showed higher levels of vigor (a positive emotion) compared to tension and depression (i.e., negative emotions), and this difference was particularly pronounced in younger adolescents. With increasing age, differences between mean levels of vigor, tension, and depression diminished. This suggests that younger adolescents showed possibly higher resilience in the first months of the pandemic. Recent COVID-19 studies, in which adolescents who were also followed prior to the pandemic, reported either no changes in negative affect, or a decrease in tension and an increase in vigor levels in comparison to pre-pandemic conditions^22,35^. Besides the well-documented vulnerabilities, adolescence is also a period characterized by goal flexibility, the capacity to quickly shift priorities, and flexibility in social behavior^2,40,41^. This flexibility enables young people to adapt adjustments in challenging situations^2^. In the current study this seemed to be primarily applicable to relatively young adolescents, and restricted to the early months of the pandemic. Indeed, other COVID-19 studies showed an increase in anxiety and depressive symptoms, and reduced life satisfaction among adolescents^42,43^. These mixed findings may be a result of the dynamic and changing effects of the pandemic, which is likely to differ from nation to nation. As a result, policy-responses to COVID-19 are continuously being altered worldwide. In addition, individual differences in pre-pandemic mood levels, coping mechanisms, and emotion-regulation might contribute to the varied findings.

In the present study we also examined the long-term effects of the pandemic. Literature on previous economic crises and long-lasting stressful events indicate that such situations tend to worsen mood and psychological wellbeing in general^28–30^. In line with our expectations, the present findings indeed indicated that the positive affect of adolescents and young adults decreased, while negative affect increased during the course of the pandemic. Interestingly, we found that particularly the younger adolescents, who at T1 still displayed greater resilience in terms of mood, experienced stronger changes in mood over time in the direction of less positive and more negative mood. Past research on adolescents’ resilience in situations of adversity have shown that their wellbeing depends on the complex interplay of systems consisting of peers, family, school, and communities, rather than individual factors alone^44^. Support from family and friends has been shown to positively influence resilient behavior^45,46^. These systems are therefore likely to contribute to the stability of young people’s resilience during the COVID-19 pandemic. As the COVID-19 pandemic continues, with unique challenges that may differ between nations, the current findings underline the need to invest in promoting resilient systems surrounding adolescents and young adults to improve their mental health^45^.

We found that adolescents with more fluctuations in all three mood states were more likely to experience family stress and inequality of opportunity in online home schooling, and that exposure to financial concerns and emotional maltreatment were positively related to depression fluctuations. Hence, we replicate findings showing associations between emotional reactivity and stressors^25,28,30^. A recent study has shown that difficulties during online home schooling and familial conflict are associated with an increase in depressive symptoms during the pandemic as compared to pre-pandemic situations^42^. In another study family income stability has been shown to be a protective factor against anxiety among Chinese college students^47^. The present findings are valuable for implementing effective interventions, as they highlight the importance of a healthy social environment and stable systems. Interventions should not only be aimed at increasing resiliency at the individual level, but also on making the systems surrounding individuals, such as households and schools, more resilient. For example, this can be achieved by providing family-based interventions aimed at improving parental skills and restoring parent–child relationships^48^. Although more complex, it is also crucial to focus on how systematic exposure to stressors, such as socioeconomic inequity, can be reduced at societal level, as it has been linked to poorer mental health among children and adolescents^49^. Re-assessing our current public health and education strategies could be one step forward. Future research should further investigate which psychological mechanisms and environmental factors may mitigate the adverse effects of COVID-19 related social and socioeconomic stressors on adolescents’ mental health, especially for vulnerable groups.

In contrast to literature on adolescents’ emotional reactivity, we did not find a peak in fluctuations in negative emotions during mid-adolescence^9–11^. Instead the present cross-sectional findings of May 2020 suggested a peak in early adulthood, possibly indicating a shift and pointing to prolonged emotional instability as a result of the current pandemic^50^. Together with our findings on the mean mood levels (i.e. less resilience with increasing age), the present study suggests that older adolescents and young adults were struggling more compared to the early-and mid-adolescents during the first months of the pandemic. A possible explanation for this could be the interference of COVID-19 with one’s transition to adulthood, which opens a new door of concerns and challenges, such as becoming an independent member of society^51^. There is one other study in which the developmental trajectories of certain mood fluctuations were not found to peak during mid-adolescence^52^. In this longitudinal study among Dutch adolescents, linear declines in sadness and anger fluctuations were found across adolescence. Hence in contrast to our results, these findings do not indicate greater emotional instability in older adolescents, making it more likely to assume that in the present study the COVID-19 pandemic may have influenced emotional instability. Maciejewski and colleagues (2015) observed an initial increase for anxiety related fluctuations during early adolescence, followed by a decrease in mid-adolescence and slight increase again at the end of adolescence, which they prescribed to the possible involvement of stress when entering adulthood^52^. The same authors reported a peak in mood fluctuations during mid-adolescence in a more recent study, however only in a minority of their sample^53^. This finding highlights the importance of examining individual differences.

The current study is novel in covering two periods and a wide age range during the pandemic. We show that as the pandemic continues to interfere with our daily lives, tension and depression fluctuations no longer peak (compared to T1) but increase linearly as a function of age. However, longitudinal data covering a longer period are needed to assess whether the pandemic indeed leads to an extension of adolescent emotional instability or whether interindividual variability in the intraindividual mood fluctuations could also play a role. We also examined whether the exposure to social and socioeconomic stressors changed over time. The experience of socioeconomic difficulties was stable, however we observed that family stress and emotional maltreatment changed. Interestingly, the latter two constructs showed an opposite pattern, as family stress decreased but emotional maltreatment increased over time. An explanation could be that due to the reopening of schools, families experienced less stress, since they spent more time independent of each other. At the same time, the pandemic was not over and other restrictions still applied. Hence in those families with more problems, the longevity of the pandemic may have outweighed the reopening of schools, resulting in more severe household hardship such as emotional maltreatment towards adolescents.

We further aimed to test the hypothesis that emotional reactivity acted as a differential susceptibility marker, resulting in more self-oriented behavior and less other-oriented behavior in an environment with more social and socioeconomic stressors. In line with our expectations emotional reactivity alone was not associated with self-or other-oriented behavior. However, contrary to our expectations, we did not find interactions with the social and socioeconomic stressors. Hence, the present study provides no evidence for the assumption of emotional reactivity as differential susceptibility marker. A possible explanation could be that in the present study we operationalized emotional reactivity, stressors, and self-oriented and other-benefitting behaviors in too many ways. The number of different variables resulted in multiple testing, making it impossible to interpret the results without proper corrections, and therefore reducing the likelihood of robust effects. Future research should target specific variables when investigating the complex interplay between emotional reactivity, self-oriented and other-benefitting behaviors, and the social environment.

The present study has several limitations which have to be taken into account when interpreting the results, and which should be addressed in future research. First, the present study has no pre-pandemic measures, which are needed to assess to what extent daily mood has changed within individuals as a function of the pandemic. Although we did not observe changes in mood levels during the pandemic, it remains unknown in the present study whether adolescents’ and young adults’ mood were different before the outbreak of COVID-19. A second limitation concerns the research sample. The samples consisted of educated participants, of whom the majority also has a medium to high socioeconomic status, which is not representative of the Dutch population. The lack of heterogeneity, in terms of educational and socioeconomic background, limits the generalizability of the present findings to the whole adolescent and young adult population. Access to participate in the study and initial eligibility could have contributed to selection bias and thus partially explain our lack of heterogeneity. Our study was done fully online and recruitment of participants was conducted through online platforms. Hence, individuals with limited access to online resources may have been missed out from our study. Our young adult sample was not representative as we focused on university college students only. In addition, adolescents from schools who did not wish to collaborate with our project did not have access to our study as they did not receive an invitation to participate. Ideally including participants who experience more variation in exposure to social and socioeconomic stressors during COVID-19 would be beneficial for our understanding of its impact on adolescents’ mood, especially as they might more severely impact adolescents with a lower socioeconomic status. As we found no differences in mood mean levels and emotional reactivity between the attrition and non-attrition group, it is unlikely that missingness for the second wave due to drop-out influenced the current findings. Lastly, the present study did not contain measures on personality or coping mechanisms, which might also have a mediating or moderating effect on the relationship between emotional reactivity and self-oriented and other-benefitting behaviors in general, as well as during the COVID-19 pandemic. Future research should include these measure as they might give us a better understanding on how to protect adolescents’ mental health during challenging times.

In conclusion, in the present study younger adolescents relative to older adolescents showed higher levels of vigor and lower levels of tension and depression in both May 2020 and November 2020. Paradoxically, the longevity of the pandemic primarily affected younger adolescents: feelings of vigor decreased, while feelings of tension and depression increased between May and November 2020, particularly among younger adolescents. We furthermore found evidence for a link between vulnerability factors (i.e. family stress and inequality of opportunity in online homeschooling) and instability in negative affect (i.e. tension and depression fluctuations) during the first months of the pandemic. Finally, together, these results highlight the complex interplay between emotional reactivity and influences from the social environment during emotional development in adolescence.

## Supplementary Information

Supplementary Information.

## Data Availability

The data generated and analysed during the present study, along with the computer code, are upon reasonable request from the authors, available in the EUR Data Repository.
