# Supplementary material for: Mood and emotional reactivity of adolescents during the COVID-19 pandemic: short-term and long-term effects and the impact of social and socioeconomic stressors
Source: Sci Rep. 2021 Jun 2;11:11563. doi: 10.1038/s41598-021-90851-x (PMC8172919; doi:10.1038/s41598-021-90851-x)
Supplement: Supplementary file 1 — Supplementary Information. [file 41598_2021_90851_MOESM1_ESM.docx]

**Mood and Emotional Reactivity of Adolescents During the COVID-19 Pandemic: Short and Long Term Effects and The Impact of Social and Socioeconomic Stressors**

Kayla H. Green*^1^, Suzanne van de Groep^1^, Sophie W. Sweijen^1^, Andrik I. Becht^12^, Moniek Buijzen^1^, Rebecca N.H. de Leeuw^3^, Danielle Remmerswaal^1^, Rianne van der Zanden^1^, Rutger C.M.E. Engels^1^, Eveline A. Crone^1^

^1^ Erasmus School of Social and Behavioural Sciences, Erasmus University Rotterdam

^2^ Research Center Adolescent Development, Utrecht University

^3^ Behavioural Science Institute, Radboud University Nijmegen

*Correspondence should be addressed to Kayla H. Green

Erasmus School of Social and Behavioural Sciences, Erasmus University Rotterdam, Burgemeester Oudlaan 50, 3000 DR Rotterdam, The Netherlands

Email:green@essb.eur.nl

**Supplementary materials**

**S1. Data preparation**

The following guidelines were followed during the data preparation in the present study. For the adolescents sample a response period of maximum 48 hours was realized, while in the student sample participants had a maximum of 36 hours to respond (1). Responses consisting of solely participant number and/or birth date were excluded (2). In case of duplicates (i.e. double responses by one individual) which both were completely filled in within 24 hours, the latter response was excluded (3). However, in case of duplicates of which one was partially completed, the incomplete response was excluded, regardless of response time (4). In case of duplicates of which one was filled in 24 hours after it was sent (e.g. a duplicate of Day 2 was filled in on Day 3) and while missing the new response of that day (Day 3), the latter response was relocated to the next day (i.e. the day on which it was filled in) (5). However, in a similar situation as described in point 4, but with the exception that the participant did fill in the new questionnaire (e.g. Day 3 filled in on Day 3), we excluded the latest response belonging to the previous questionnaire (duplicate of Day 2 filled in on Day 3) (6). When a questionnaire was filled in 24 hours after it was sent and after filling in the new questionnaire, the daily responses of that questionnaire were excluded. Weekly measures were not removed (7). When a questionnaire was filled in 24 hours after it was sent and no duplicate was present, and the new questionnaire of that day was not filled in (e.g. Day 2 filled in on Day 3, and Day 3 missing), the questionnaire was still excluded as it would have been unclear about which day the questions were filled in (8). In case of two incomplete responses which both were filled in within 24 hours, the latter response was excluded (9). These preparatory measures were identical for the two cohorts.

**S2. Aim 1: Details regarding assumption checks**

Table S2a.  *Overview on the kurtosis and skeweness checks on all variables from Aim 1.*

|  | Kurtosis | Skeweness | Min | Max |
| --- | --- | --- | --- | --- |
| **Mood levels** |  |  |  |  |
| Vigor_T1_ | -.62 | -.17 | 1.04 | 4.94 |
| Tension_T1_ | 1.61 | 1.39 | 1.00 | 4.83 |
| Depression_T1_ | 3.13 | 1.78 | 1.00 | 4.75 |
| Vigor_T2_ | -.52 | -.21 | 1.04 | 4.83 |
| Tension_T2_ | 1.68 | 1.33 | 1.00 | 4.95 |
| Depression_T2_ | 2.85 | 1.65 | 1.00 | 4.61 |
| **Mood fluctuations** |  |  |  |  |
| Vigor_T1_ | .45 | .31 | .20 | 1.93 |
| Tension_T1_ | -.51 | .09 | .00 | 1.61 |
| Depression_T1_ | -.46 | .33 | .00 | 1.66 |
| Vigor_T2_ | .69 | .29 | .00 | 1.95 |
| Tension_T2_ | -.56 | -.06 | .00 | 1.53 |
| Depression_T2_ | -.65 | ,29 | .00 | 1.59 |

Note: the minimum and maximum scores displayed in the table represent actual responses, and therefore do not show the complete response range of the measures.

*Sphericity test: analyses first wave*

Mauchly’ test indicated that the assumption of sphericity was violated, (*χ^2^* (2) = 1056.45, *p* < .001) in the first RM ANOVA (i.e. mood level differences T1 with age as linear factor) of Study 1. In the second RM ANOVA of study 1 (addition of age as quadratic factor to the model), the assumption of sphericity was again violated, *χ^2^* (2) = 1043.64, *p* < .001. Therefore, we reported the Greenhouse-Geisser corrected test (respectively, *ε* = .58 and *ε* = .58).

Mauchly’ test indicated that the assumption of sphericity was violated, (*χ^2^* (2) = 159.57, *p* < .001) in the third RM ANOVA (i.e. mood fluctuation differences T1 with age as linear factor) of Study 1. In contrast to the previous RM ANOVAs, here the estimate of sphericity was above .75 (*ε* = .85). Hence, we used the Huyn-Feldt corrected test for the second RM ANOVA instead of the Greenhouse-Geisser estimate ^54^. Mauchly’ test indicated that the assumption of sphericity was also violated, (*χ^2^* (2) = 150.88, *p* < .001) when adding quadratic age to the model (*ε* = .85). In line with the abovementioned analysis, we reported the Huyn-Feldt corrected test here as well.

*Sphericity test: analyses second wave*

Mauchly’ test indicated that the assumption of sphericity was violated, (*χ^2^* (2) = 613.75, *p* < .001) in the first RM ANOVA (i.e. mood level differences T2 with age as linear factor) of Study 1. In the second RM ANOVA of study 1 (addition of age as quadratic factor to the model), the assumption of sphericity was again violated, *χ^2^* (2) = 607.01, *p* < .001. Therefore, we reported the Greenhouse-Geisser corrected test (respectively, *ε* = .58 and *ε* = .58).

Mauchly’ test indicated that the assumption of sphericity was violated, (*χ^2^* (2) = 71.82, *p* < .001) in the third RM ANOVA (i.e. mood fluctuation differences T2 with age as linear factor). Here the estimate of sphericity was above .75 (*ε* = .88). Hence, we used the Huyn-Feldt corrected test for the second RM ANOVA instead of the Greenhouse-Geisser estimate ^54^

*Sphericity test: long-term effects*

Mauchly’ test indicated that the assumption of sphericity was violated, *χ^2^* (2) = 395.27 *p* < .001 (interaction effect mood state x time). Therefore, we reported the Greenhouse-Geisser corrected test (respectively, *ε* = .63) in the RM ANOVA regarding the long-term effects on mood levels (Study 1).

Table S2b. *Bivariate correlations between mood levels and mood fluctuations in May 2020 (T1)*

|  | **Mood levels** | | | **Mood fluctuations** | | |
| --- | --- | --- | --- | --- | --- | --- |
|  | Vigor | Tension | Depression | Vigor | Tension | Depression |
| **Mood levels** |  |  |  |  |  |  |
| Vigor | 1 | -.41*** | -.44*** | -.09** | -.41*** | -.50*** |
| Tension |  | 1 | .84*** | -.01 | .65** | .66** |
| Depression |  |  | 1 | .01 | .52*** | .73*** |
| **Mood fluctuations** |  |  |  |  |  |  |
| Vigor |  |  |  | 1 | .20*** | .19*** |
| Tension |  |  |  |  | 1 | .73*** |
| Depression |  |  |  |  |  | 1 |

**p* < .05, ***p* < .01, ****p* < .001

Table S2c. *Bivariate correlations between mood levels and mood fluctuations in November 2020 (T2).*

|  | **Mood levels** | | | **Mood fluctuations** | | |
| --- | --- | --- | --- | --- | --- | --- |
|  | Vigor | Tension | Depression | Vigor | Tension | Depression |
| **Mood levels** |  |  |  |  |  |  |
| Vigor | 1 | -.40*** | -.45*** | -.10* | -.38*** | -.51*** |
| Tension |  | 1 | .86*** | -.10* | .57*** | .62*** |
| Depression |  |  | 1 | .09* | .47*** | .71*** |
| **Mood fluctuations** |  |  |  |  |  |  |
| Vigor |  |  |  | 1 | .32*** | .28*** |
| Tension |  |  |  |  | 1 | .71*** |
| Depression |  |  |  |  |  | 1 |

**p* < .05, ***p* < .01, ****p* < .001

**S3. Aim 2: Details regarding assumption checks**

Table S3a.  *Overview on the kurtosis and skeweness checks on additional variables from Aim 2.*

|  | Kurtosis | Skeweness | Min | Max |
| --- | --- | --- | --- | --- |
| **Social stressors** |  |  |  |  |
| Family stress | -.54 | .55 | 1.00 | 7.00 |
| Emotional maltreatmemt | 1.42 | 1.25 | 1.00 | 4.50 |
| **Socioeconomic stressors** |  |  |  |  |
| Inequality of opportunities | 2.04 | 1.36 | 1.00 | 4.80 |
| Financial concerns | 1.33 | 1.28 | 1.00 | 5.00 |
| **Self-oriented behavior** |  |  |  |  |
| Unimportance rules | .15 | .71 | 1 | 6.67 |
| Disobeying the rules | -.64 | .43 | 0 | 2 |
| **Other-oriented behavior** |  |  |  |  |
| Emotional support to family | .16 | .70 | 0 | 5 |
| Emotional support to friends | -.61 | .02 | 0 | 5 |
| Helping others during COVID-19 | -.24 | -.42 | 1 | 6.6 |

Table S3b. *Bivariate correlations among all variables related to Aim 2.*

|  | **Mood fluctuations** | | | **Social stressors** | | **Socioeconomic stressors** | | **Self-oriented behavior** | | **Other-benefitting behavior** | | |
| --- | --- | --- | --- | --- | --- | --- | --- | --- | --- | --- | --- | --- |
|  | Vigor | Tension | Depression | Family stress | Emotional maltreatment | Inequality of opportunity | Financial concerns | Unimportance rules | Disobeying the rules | Emotional support to family | Emotional support to friends | Helping others |
| Vigor | 1 | .22*** | .24*** | .11* | .06 | .13* | .05 | .07 | .09 | -.06 | -.02 | -.01 |
| Tension |  | 1 | .75*** | .16** | .07 | .10* | .08 | .04 | .02 | -.00 | .10* | -.05 |
| Depression |  |  | 1 | .19*** | .12* | .19*** | .12* | .03 | -.01 | -.02 | .03 | -.01 |
| Family stress |  |  |  | 1 | .51*** | .42*** | .34*** | .13** | .11* | -.08 | .02 | -.02 |
| Emotional maltreatment |  |  |  |  | 1 | .50*** | .30*** | .08 | .00 | -.25*** | -.10* | -.12** |
| Inequality of opportunity |  |  |  |  |  | 1 | .40*** | .16*** | .10* | -.15** | -.10* | -.00 |
| Financial concerns |  |  |  |  |  |  | 1 | .08 | .06 | .01 | .06 | .01 |
| Unimportance rules |  |  |  |  |  |  |  | 1 | .33*** | -.06 | .04 | .06 |
| Disobeying the rules |  |  |  |  |  |  |  |  | 1 | -.05 | .10* | .05 |
| Emotional support to family |  |  |  |  |  |  |  |  |  | 1 | .61*** | .40*** |
| Emotional support to friends |  |  |  |  |  |  |  |  |  |  | 1 | .34*** |
| Helping others |  |  |  |  |  |  |  |  |  |  |  | 1 |

Note: Associations with one of the three mood fluctuations measurements were all controlled for mood level, except for the correlations among the mood states themselves.

Inequality of opportunities = Inequality of opportunities in online homeschooling, Unimportance rules = Perceived unimportance of the rules, Helping others = Helping others during the COVID-19 pandemic.

**p* < .05, ***p* < .01, ****p* < .001

**S4. Aim 2: Main effects social and socioeconomic stressors on self-oriented and other-benefitting behaviors***Associations between stressors and self-oriented and other-benefitting behaviors*

We performed five stepwise regression analyses to assess the assumption that exposure to social and socioeconomic stressors is associated with more self-oriented behavior and less other-oriented behavior. Social stressor and socioeconomic stressors were entered as independent variables to the model. Since the independent variables were composite scores made out of z-scores, mean centering was not needed as extra combat step against multicollinearity ^34^. The dependent variables were: 1) the perceived unimportance of the rules, 2) disobeying the rules, 3) the opportunity to prove emotional support to family, 4) and friends, and 5) the willingness to help others during COVID-19.

In the first stepwise regression analysis we tested the assumption that social and socioeconomic stressors have a main effect on adolescents’ perceived unimportance of the COVID-19 rules. The socioeconomic stressor accounted a significant amount of the variance in the perceived unimportance of the COVID-19 rules, and ended up being the only independent variable in the model 1 (*R^2^ =* .03, *F*(1 , 445) = 11.04 , *p =* .001). Dealing with exposure to socioeconomic stress was positively associated with the perceived unimportance of the COVID-19 rules (*b* = .29, *SE B* = .09, β = .16, *p* = .001). Adding social stressor, did not result in an increase in the explained variance for adolescents’ perceived unimportance of the COVID-19 rules  *(p* > .05).

A second stepwise regression analysis was performed to assess whether social and socioeconomic stressors partly account for the variance in disobeying the COVID-19 rules. Results revealed that the socioeconomic stressors composite score was again the only independent variable in the model, which accounted a significant amount of the variance in disobeying the COVID-19 rules (*R^2^ =* .01, *F*(1 , 445) = 4.54 , *p* = .034). Dealing with socioeconomic stress was positively related to disobeying the COVID-19 rules (*b* = .08, *SE B* = .04, β = .10, *p* = .034). Exposure to the social stressor composite score, did not result in an increase in the explained variance disobeying the COVID-19 rules (*p* > .05).

Next we tested whether the variance in the opportunity to provide emotional support to family was partially depended on the exposure to social and socioeconomic stressors. Results showed that the first model, in which the social stressor composite score was the only independent variable, accounted a significant amount of variance (*R^2^ =* .05, *F*(1 , 446) = 23.09 , *p* < .001). Dealing with exposure to social stress within the family context was negatively associated with emotional support to family (*b* = -.37, *SE B* = .08, β = -.22, *p* < .001). The socioeconomic stressor composite score did not significantly increase the explained variance in dependent variable (*ps* > .05).

In the fourth stepwise regression analysis we assessed whether there was a main effect of social and socioeconomic stressors on the opportunity provide emotional support to friends. None of the independent variables accounted a significant amount of the variance in the willingness to help other during COVID-19.

Lastly, we tested whether the variance in the willingness to help others during COVID-19 could partially be accounted for by the different social and socioeconomic stressors. In the final and only model, the independent variable, social stress within the family context, accounted a significant amount of the variance in the willingness to help other during COVID-19 (*R^2^ = .*01, *F*(1, 446) = 5.06 , *p* = .025). Exposure to the social stressor composite score was negatively associated with the willingness to help others during COVID-19 (*b* = -.20, *SE B* = .09, β = -.11, *p* =.025). Adding the socioeconomic stressors composite score to the model did not lead to an increase in the explained variance in the willingness to help others during COVID-19 (*p* > .05).

*
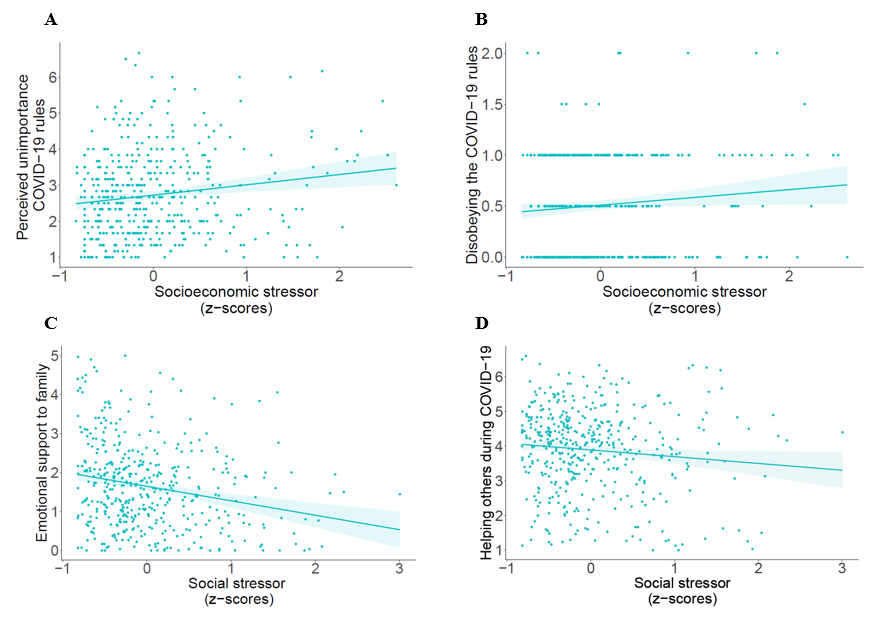
*

*Figure S4.* These scatterplots show the main effects of social and socioeconomic stressors on self-and other-oriented behavior. Graph A illustrates the positive association between the socioeconomic stressor composite score and the perceived unimportance of the COVID-19 rules at T1 (*p* = .001). Graph B shows the positive association between the socioeconomic composite score and disobeying the COVID-19 rules (*p* <.05). Next is Graph C, which illustrates the negative association between the social stressor composite score and the opportunity for emotional support to family (*p* < .001). Graph D shows the negative association between the social stressor composite score and helping others during COVID-19 (*p* < .05). Note: the social stressor is a composite score of the measures emotional maltreatment and family stress, the socioeconomic stressor is a composite score of the measures inequality of opportunity in online home schooling and financial concerns.

**S5. Aim 2: Details on non-significant findings moderation analyses**

Table S5a. *Overview of the non-significant findings from the moderation analyses, specifically for self-oriented behaviors.*

|  | ***t*** | ***b*** | ***SE*** | ***p*** |
| --- | --- | --- | --- | --- |
| **Perceived unimportance COVID-19 rules** |  |  |  |  |
| Vigor fluctuations x social stressor | 1.43 | .47 | .33 | .154 |
| Tension fluctuations x social stressor | -1.47 | -.34 | .23 | .144 |
| Depression fluctuations x social stressor | -1.79 | -.41 | .23 | .075 |
| Vigor fluctuations x socioeconomic stressor | 1.66 | .51 | .31 | .100 |
| Tension fluctuations x socioeconomic stressor | -.24 | -/06 | .24 | .810 |
| Depression fluctuations x socioeconomic stressor | -.83 | -.20 | .24 | .408 |
| **Disobeying the COVID-19 rules** |  |  |  |  |
| Vigor fluctuations x social stressor | .83 | .12 | .14 | .409 |
| Tension fluctuations x social stressor | 1.53 | .15 | .10 | .127 |
| Depression fluctuations x social stressor | 1.02 | .10 | .10 | .308 |
| Vigor fluctuations x socioeconomic stressor | 1.18 | .15 | .13 | .238 |
| Tension fluctuations x socioeconomic stressor | 1.50 | .15 | .10 | .134 |
| Depression fluctuations x socioeconomic stressor | -.19 | -.02 | .10 | .851 |

Note: the social stressor is a composite score of the measures emotional maltreatment and family stress, the socioeconomic stressor is a composite score of the measures inequality of opportunity in online homeschooling and financial concerns

Table S5b**.** *Overview of the non-significant findings from the moderation analyses, specifically for other-benefitting behaviors.*

|  | ***t*** | ***b*** | ***SE*** | ***p*** |
| --- | --- | --- | --- | --- |
| **Emotional support to family** |  |  |  |  |
| Vigor fluctuations x social stressor | 1.57 | .46 | .30 | .118 |
| Tension fluctuations x social stressor | -.55 | -.11 | .21 | .583 |
| Depression fluctuations x family stressor | .45 | .09 | .21 | .653 |
| Vigor fluctuations x socioeconomic stressor | 1.37 | .39 | .28 | .171 |
| Tension fluctuations x socioeconomic stressor | -.93 | -.21 | .23 | .354 |
| Depression fluctuations x socioeconomic stressor | .65 | .15 | .23 | .518 |
| **Emotional support to friends** |  |  |  |  |
| Tension fluctuations x social stressor | 1.57 | .33 | .21 | .116 |
| Depression fluctuations x social stressor | 1.63 | .35 | .21 | .104 |
| Vigor fluctuations x socioeconomic stressor | .21 | .06 | .29 | .835 |
| Tension fluctuations x socioeconomic stressor | .38 | .09 | .22 | .703 |
| Depression fluctuations x socioeconomic stressor | -.13 | -.03 | .23 | .893 |
| **Helping others during COVID-19** |  |  |  |  |
| Depression fluctuations x social stressor | -1.17 | -.27 | .23 | .243 |
| Tension fluctuations x socioeconomic stressor | -.85 | -.21 | .25 | .396 |
| Depression fluctuations x socioeconomic stressor | -.47 | -.12 | .25 | .635 |

Note: the social stressor is a composite score of the measures emotional maltreatment and family stress, the socioeconomic stressor is a composite score of the measures inequality of opportunity in online home schooling and financial concerns.
